# Acute Safety and Efficacy of Intranasal Esketamine Spray Plus an Oral Antidepressant in Patient With Treatment-Resistant Depression From a University Hospital in Korea

**DOI:** 10.1192/bjo.2025.10798

**Published:** 2025-06-20

**Authors:** Sang-Yeol Lee, Kyung-Joon Min, Chan-Mo Yang

**Affiliations:** 1 Department of Psychiatry, Wonkwang University School of Medicine and Hospital, Iksan, Korea, Republic of; 2 Department of Psychiatry, College of Medicine, Chung-Ang University, Seoul, Korea, Republic of

## Abstract

**Aims:** There is limited real-world evidence for patients with treatment-resistant depression (TRD) receiving esketamine nasal spray in Korea. This study is aimed to evaluate the acute safety and efficacy of intranasal esketamine in patients with TRD from a university hospital in Korea.

**Methods:** This open-label and prospective study used data collected from the Wonkwang University Hospital. Patients with TRD received esketamine plus an oral antidepressant during the treatment period. This study comprised a 4-week screening, 2-week induction and 2-week follow-up.

**Results:** A total of 22 TRD patients received esketamine April 2021–April 2023. This group was predominantly female, and have several psychiatric comorbidities and high exposure to psychiatric medications. We observed significant reductions (p<0.001) in average Hamilton Depression Rating Scale (HAM-D) and Clinical Global Impression severity (CGI-S) from baseline (HAM-D: 25.6+5.3, CGI-S: 5.1+1.5) to last available treatment(HAM-D: 19.6+3.9, CGI-S: 3.0+1.2). Suicidal thought also significantly reduced from baseline(2.33+0.7) to last treatment (1.37+0.6)(p<0.001).

Compared with the baseline, 23% of HAM-D, 43% of CGI-S and 42% of suicidal thought of TRD patients reduced in the 4 weeks. There were no reports of serious adverse events.

**Conclusion:** The acute safety and efficacy of esketamine in the Korean patients was generally consistent with the international studies. There was no new safety signal and no indication for abuse. Acute efficacy occurred early during the induction phase.

